# Immobilization of *Staphylococcus aureus* Sortase A on Chitosan Particles and Its Applications in Peptide-to-Peptide Ligation and Peptide Cyclization

**DOI:** 10.3390/molecules23010192

**Published:** 2018-01-19

**Authors:** Min Yang, Haofei Hong, Shaozhong Liu, Xinrui Zhao, Zhimeng Wu

**Affiliations:** Key Laboratory of Carbohydrate Chemistry & Biotechnology, Ministry of Education, School of Biotechnology, Jiangnan University, Wuxi 214122, China; 18262281871@163.com (M.Y.); hhf_1992@163.com (H.H.); liushaozhong90@163.com (S.L.); zhaoxinruibs0511@163.com (X.Z.)

**Keywords:** sortase A, chitosan particle, immobilization, ligation, cyclization

## Abstract

Chitosan macro-particles prepared by the neutralization method were applied to Sortase A (SrtA) immobilization using glutaraldehyde as a crosslinking agent. The particles were characterized by Fourier transform infrared spectroscopy (FTIR) and scanning electron microscopy (SEM). Response surface methodology (RSM) was employed to optimize the immobilization process. An average specific activity of 3142 U (mg protein)^−1^ was obtained under optimized immobilization conditions (chitosan concentration 3%, SrtA concentration 0.5 mg·mL^−1^, glutaraldehyde concentration 0.5%, crosslinking and immobilization at 20 °C, crosslinking for 3 h, and an immobilization time of 8 h). The transpeptidase activity of immobilized SrtA was proved by a peptide-to-peptide ligation with a conversion yield approximately at 80%, and the immobilized catalyst was successfully reused for five cycles without obvious activity loss. Moreover, the scale-up capability of using immobilized SrtA to catalyze a head-to-tail peptide cyclization was investigated in a batch reaction and the conversion yield was more than 95% when using 20 mg of peptide as a substrate.

## 1. Introduction

Sortase A (SrtA, EC 3.4.22.70) is a transpeptidase derived from *Staphylococcus aureus*. It can recognize the Leu-Pro-X-Thr-Gly (LPXTG, X standing for any amino acid except cysteine) pentapeptide sequence at the C-terminus of protein and cleave the amide bond between threonine and glycine residues to form an acyl-enzyme complex, which subsequently reacts with nucleophiles containing oligo-glycine to generate a ligation product [1,2,3]. In recent years, SrtA has been widely utilized in the modification of biomolecules, including protein–protein fusion [4,5,6,7,8], in vitro and in vivo protein functionalization [9,10], peptide and protein cyclization [11,12,13], and cell and biomaterial surface functionalization [14,15,16,17,18] among others [19,20,21,22]. SrtA-mediated ligation was applied to prepare an antibody-drug conjugate for a therapeutic purpose as well [23,24]. Therefore, SrtA is a highly valuable biocatalyst for biomolecule engineering.

Currently, enzyme SrtA is readily accessed by using recombinant expression technology in *Escherichia coli* [25]. Enzyme evolution technology has generated enhanced SrtA with improved catalytic activity and specificity [26,27]. However, several bottlenecks have hindered its future industrial application. Although it has been reported that the expression yield of SrtA in *E. coli* was optimized to a level of approximately 232 mg·L^−1^ [28], it was reported that the enzyme and substrates are used at a near-equimolar concentration in some cases. Thus, the disposable use of free SrtA as a biocatalyst is a waste of a resource, especially in a large-scale reaction.

The use of immobilized enzymes in industrial processes, in comparison with the use of soluble enzymes, could reduce process costs by reducing the quantity of enzyme required, as well as simplifying the downstream purification of the products, since the immobilized biocatalyst can be recovered and reused easily as long as the enzyme remains active for several reaction cycles. In addition, a proper immobilization method may improve enzyme stability and also some other parameters, such as activity and selectivity [29,30,31].

To address these issues, several strategies have recently been developed to immobilize SrtA onto a solid support for the site-specific modification of proteins. Beck-Sickinger et al. firstly explored the immobilization of SrtA onto PEGA resin (polyethylene glycol polyacrylamide copolymer resin) by combining the expressed protein ligation and click chemistry. This approach generated relatively low enzymatic activity after immobilization, as the conversion yield is only 10% in peptide-to-peptide ligation [32]. Recently, Pentelute’s group immobilized evolved SrtA onto Ni-NTA agarose particles via the His-tag, which was successfully applied in flow-based enzymatic ligation [33]. We have developed a one-step purification and immobilization strategy for the purification and immobilization of His-tag SrtA onto an Ni-modified magnetic particle by using the fermentation supernatant directly. The immobilized SrtA was proved to retain full enzymatic activity in chemoselective ligation with excellent reusability without obvious activity loss [34]. However, these approaches relied on using expensive nickel-modified particles, which is not suitable for industrial application in practice. Ploegh and coworkers investigated chemically immobilizing SrtA onto cyanogen bromide-activated Sepharose beads and applied it in flow- and batch-based protein C- or N-terminus labeling [35]. However, this randomly oriented immobilization resulted in decreased enzyme activity probably because the immobilization process is not optimized. In addition, the enzymatic profiles of the immobilized SrtA were not studied. Most recently, Francis’s group site-specifically modified the N-terminus of evolved SrtA with lithocholic acid and demonstrated an affinity-based approach to capture and recycle SrtA with a β CD-modified sepharose resin. The disadvantage is that preparing a lithocholic acid–SrtA conjugate is complicated, which requires three more steps in a moderate yield [36]. Thus, to explore immobilized SrtA for future industrial application, searching for an economical and a biocompatible carrier for SrtA as well as a simple immobilization procedure is highly desired.

Chitosan is an abundant deacetylated product of natural polymer chitin. Because of its desirable characteristics, such as a high affinity to proteins, the availability of reactive functional groups to couple with enzymes, hydrophilicity, mechanical stability and rigidity, ease of preparation, and cost-effectiveness, chitosan is widely used as a support material for many enzyme immobilizations [37,38,39,40]. Moreover, chitosan is a nontoxic, biocompatible, and biodegradable material, which can be used safely in food, pharmaceutic, and agricultural applications [41,42,43,44]. In this work, we explored chitosan as a carrier for SrtA immobilization. Specifically, the covalent immobilization of SrtA onto chitosan particles was achieved using glutaraldehyde as a crosslinking reagent [45] via a shiff-base bond. As reducing the shiff-base bond to a secondary amine had no significant improvement on enzyme stability and other characteristics, no reducing reagent was applied in the SrtA immobilization procedure, which also avoided potentially deleterious effects on the enzymes’ structures, the disulphide bond, and the peptide bond [46]. Addtionally, the process was optimized by response surface methodology (RSM) [47]. The immobilized SrtA was further characterized by Fourier transform infrared (FTIR) spectroscopy and scanning electron microscopy (SEM). The physical-chemical properties of the immobilized SrtA as well as the application of the immobilized SrtA in peptide-to-peptide ligation were studied. Finally, the scale-up capability of the immobilized SrtA to catalyze a head-to-tail peptide cyclization reaction was investigated in a batch reaction using up to 20 mg of peptide as a substrate.

## 2. Results and Discussion

### 2.1. Characterization of SrtA Immobilized Chitosan Particles

**Figure 1:**
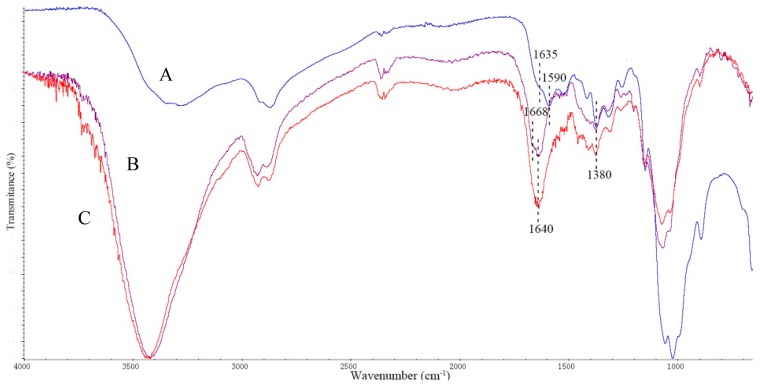
FTIR spectra of (**A**) pure chitosan particles; (**B**) chitosan particles treated with glutaraldehyde; and (**C**) chitosan particles with immobilized SrtA.

FTIR was used to characterize chitosan particles, chitosan particles crosslinked with glutaraldehyde, and chitosan particles with immobilized SrtA. The FTIR spectra are presented in Figure 1. The broad band between 3100 and 3700 cm^−1^ is the O-H stretching vibration, mainly from water, which overlaps with the amine stretching vibrations (N-H) in the same region. The bands between 2800 and 3000 cm^−1^ were attributed to the C-H stretching vibration. The absorption bands between 1000 and 1100 cm^−1^ were related to C-O and C-N stretching vibrations and C-C-N bending vibrations. Peaks at 1380 cm^−1^ correspond to a –C-O-H stretching vibration of a primary –OH group in the chitosan structure. For bare chitosan particles (Figure 1A), the IR spectra show characteristics of the N-H bending vibration of primary amines present on the chitosan structure with absorptions at 1590 cm^−1^ and 1635 cm^−1^. After activation by glutaraldehyde as shown in Figure 1B, the IR spectra showed a weak shoulder adsorption at 1668 cm^−1^ and a strong peak at 1640 cm^−1^, indicating the presence of the second free aldehyde group from glutaraldehyde and the formation of imine bonds (C=N), respectively [48]. It is worth noting that after the glutaraldehyde-activated chitosan beads were treated with enzyme SrtA and subsequently quenched with alanine, the aldehyde shoulder peak disappeared, which demonstrated that SrtA was successfully immobilized onto chitosan beads.

**Figure 2:**
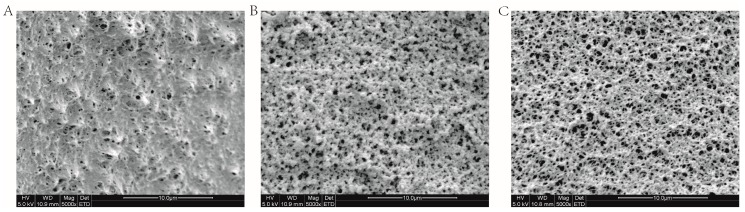
SEM images of (**A**) pure chitosan particles; (**B**) glutaraldehyde activated particles; and (**C**) Sortase A (SrtA) immobilized particles.

The surface morphology of the three types of particles was observed by scanning electron microscopy (SEM) (Figure 2). As shown in Figure 2A, bare chitosan particles presented an uneven surface with many randomly distributed large pores. After the chitosan particles were activated by glutaraldehyde and subsequently coupled with SrtA, the surface structure became relatively smoother and tighter and showed a rough fibrous network (Figure 2B,C). These morphology changes should be the results of grafting glutaraldehyde chains and the attachment of SrtA [49].

### 2.2. Process Optimization of Immobilization of SrtA onto Chitosan Particles

#### 2.2.1. Identification of the Significant Variables Using the Plackett–Burman (PB) Design

To screen for the most significant factors that influence SrtA immobilization, seven factors that might affect SrtA immobilization on chitosan particles were examined by PB design using a set of 12 runs. The PB design is based on the first-order model given below:

$$Y=\beta_{0}+\Sigma \beta_{i}X_{i}$$

where Y is the response (specific activity of immobilized SrtA), $\beta_{0}$ is the model coefficient, $\beta_{i}$ is the linear coefficient, and $X_{i}$ is the set of variables investigated. The main effect of variables, *F*, and *p*-values generated by Design Expert@ 8.0.6 are presented in Table 1.

A main effect figure with a positive sign indicates that the high value of the corresponding variable is near optimum and a negative sign indicates that the low value of this variable is close to optimum [47]. The *F* value (20.91) of the model was found to be significant. A *p*-value <0.05 indicated that model terms are significant. As shown in Table 1, two negative effects and one positive effect, including concentration of glutaraldehyde, concentration of SrtA, and immobilization time, were the three most significant factors affecting SrtA immobilization. A declaration on the other variables was done according to whether the corresponding effect was positive or negative. Thus, the concentration of chitosan (3%), crosslinking temperature (20 °C), immobilization temperature (20 °C), and crosslinking time (3 h) were chosen for the next experiment.

#### 2.2.2. Optimization of Operational Parameters by the Box–Behnken Experimental Design (BBD)

The most significant operational parameters, including concentration of glutaraldehyde, concentration of SrtA, and immobilization time, that were obtained from the PB design for SrtA immobilization on chitosan were further optimized by the BBD of RSM. The coded and uncoded levels of the three independent variables are listed in Table 2. Table 3 shows the experimental design and the response data obtained from the 17 trial runs. The final equation in terms of coded factors for the specific activity of immobilized SrtA was as follows:

Y = 3140.58 + 261.49A + 11.41B − 283.36C + 75.13AB − 119.07AC + 430.99BC − 1407.44A^2^ − 239.31B^2^ − 335.08C^2^

where Y is the response of specific activity and A, B, and C, are the coded values of the test variables (concentration of glutaraldehyde, concentration of SrtA, and immobilization time), respectively. The regression analysis and ANOVA are shown in Table 4. The *F* value (27.75) implied the model to be significant and values of Prob > *F* (0.0001) less than 0.05 were considered significant. The “Lack of Fit *F*-value” of 1.25 implies the Lack of Fit is not significant relative to pure error.

**Figure 3:**
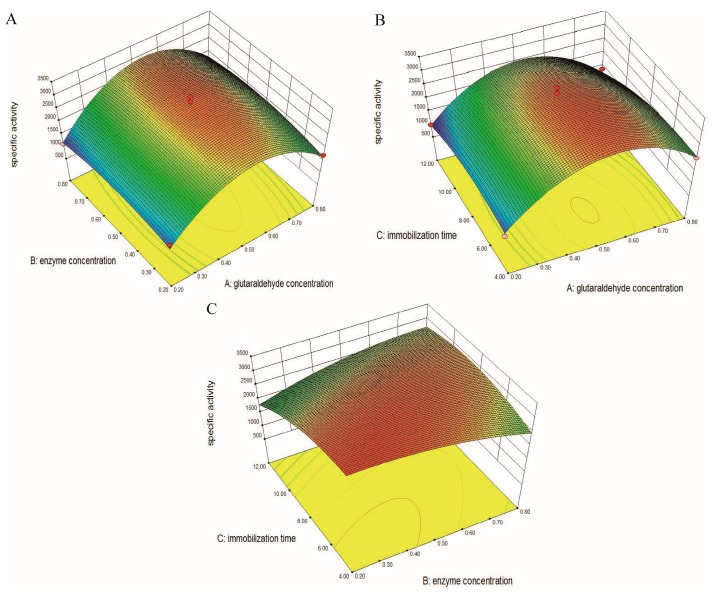
Three-dimensional plots for the interaction effects of A and B (**A**); A and C (**B**); and B and C (**C**) on specific activity.

The interactive effects of the above three factors on the specific activity are represented in Figure 3. The three-dimensional (3D) plot (Figure 3A) between concentration of glutaraldehyde (A) and concentration of the enzyme (B) is dome-shaped, indicating maximum specific activity near the mid values of both the components. The analysis of the interaction between concentration of glutaraldehyde (A) and immobilization time (C) (Figure 3B) shows maximum specific activity at a concentration of glutaraldehyde in the middle range of ‘−1’ and ‘+1’ level and an immobilization time slightly close to ‘−1’ level. It is also observed in Figure 3C that there is no significant change in the specific activity of immobilized SrtA with a concentration of enzyme (B) ranging from 0.2 to 0.6 mg·mL^−1^ and an immobilization time (C) ranging from 4 to 9 h. Furthermore, a high level of both of the two components will result in a decline in specific activity.

Based on the regression model and predictions by a software design expert, a maximum specific activity value of 3290.44 U·mg^−1^ of immobilized SrtA can be achieved in the conditions of 0.53% glutaraldehyde, 0.25 mg·mL^−1^ SrtA, and 4.04 h of immobilization time. To verify this model, SrtA immobilization was performed under these predicted optimal conditions and a specific activity of 3242.40 U·mg^−1^ of immobilized SrtA was obtained with an activity retention (AR) at 10.43% (Table 3, entry 9). However, it is worth noting that the total enzymatic activity of immobilized SrtA in these conditions was relatively low (276.50 U), which may result in a low conversion yield in a practical application of SrtA-mediated ligation. It is interesting to observe that another five center points of immobilization exhibited significant total enzymatic activity (588.28 U, 550.10 U, 637.48 U, 592.40 U, and 572.47 U), while the specific activity only decreases marginally (Table 3, entry 2, 7, 10, 16, and 17). Therefore, to maintain maximum total enzymatic activity and specific activity, the optimum immobilization conditions were chosen as: 3% chitosan, 0.5% glutaraldehyde, 0.5 mg·mL^−1^ SrtA, crosslinking and immobilization at 20 °C, crosslinking for 3 h, and immobilization for 8 h. As for the relatively low specific activity retention, it may be attributed to the enzyme conformation integrity alteration and a less-favorable mass diffusion after immobilization. Addtionally, this phenomenon is often observed in enzyme immobilization using chitosan particles as a solid support [37,42]. The underlying mechanisms are complicated and elusive and further investigations are needed.

### 2.3. pH and Thermo-Stability of Immobilized SrtA

**Figure 4:**
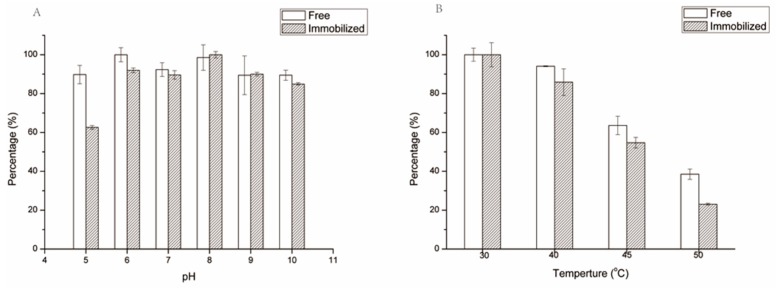
pH (**A**) and thermo- (**B**) stability of free and immobilized SrtA. Experiments were performed as described in Section 3.5.

The pH and thermo-stability are important properties of an immobilized enzyme for industrial application. The pH stability of free and immobilized SrtA was investigated by incubating the two types of SrtA in buffers at 25 °C for 30 min with different pH values. As shown in Figure 4A, immobilized SrtA exhibited similar pH stability to free enzyme at a pH range of 7 to 10. For example, under an optimal pH of 8, the immobilized SrtA retained the full enzyme activity without activity loss. It was found that both of them reserved more than 80% of enzyme activities at pH 7, 9, and 10, indicating the excellent pH stability of immobilized SrtA. However, immobilized SrtA is less stable than free SrtA under an acidic condition, such as a pH at 5 and 6. It is speculated that this is because the physical-chemical properties of the chitosan particles were altered, which affects the pH stability of immobilized SrtA.

The thermo-stability of immobilized SrtA was examined by incubating it at 30, 40, 45, and 50 °C for 30 min in buffers at a pH of 8.0. As illustrated in Figure 4B, the immobilized SrtA retained full enzyme activity at 30 degrees. When the immobilized SrtA was incubated at 40 degrees, it still retained 90% of its enzyme activity. However, when the temperature was increased to 45 and 50 degrees, the enzyme activities of the immobilized SrtA dropped to 54.7% and 23.1%, respectively. For the free SrtA, its enzyme activities were also reduced to 63.6% and 38.5% at 45 and 50 degrees, respectively. This result indicated that the attachment of SrtA onto the chitosan surface had no improvement effect for its thermal stability. This phenomenon may be caused by the change of enzyme conformation because of strong random multipoint covalent interaction between the enzyme and the chitosan carrier, which reduced the enzyme’s operational stability [50,51].

### 2.4. Immobilized SrtA-Mediated Peptide-to-Peptide Ligation

**Figure 5:**
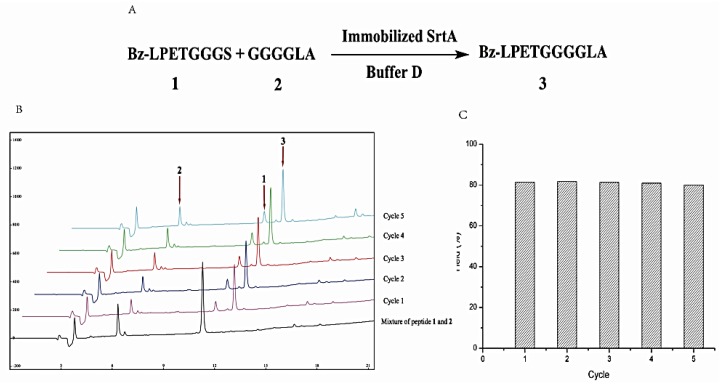
(**A**) Immobilized SrtA-mediated ligation of peptide **1** and **2**; (**B**) HPLC diagraph of immobilized SrtA-mediated ligation of peptide **1** and **2**; (**C**) Reuse and recycling of immobilized SrtA-mediated ligation of peptide **1** and **2**. Experiments have been performed in pH 7.5 at 37 °C for 1 h with ten chitosan-immobilized SrtA beads. Other specifications are described in Section 3.6.1.

To examine the transpeptidation activity of immobilized SrtA, donor peptide **1** and acceptor **2** were used as a model reaction (Figure 5). The reaction mixture was incubated with ten chitosan particles in 200 μL at 37 °C and monitored by HPLC and MALDI-TOF MS. As shown in the HPLC profile (Figure 5B), a new peak was generated at a retention time of 12.35 min in 1 h, which was confirmed to be the ligation product **3** (MS_cal_: 975.0, MS_obv_: 997.4 [M + Na]^+^) by MALDI-TOF MS (SI, Figure S1). The conversion yield was 80% based on the peak area. The reusability of immobilized SrtA was assessed as well. Thus, after the reaction was finished, SrtA-immobilized chitosan particles were recovered followed by washing with buffer B. Following the same protocol, the SrtA immobilized chitosan particles were recovered and reused for another four cycles. As presented in Figure 5C, the ligation efficiency was very stable in these five cycles, indicating that the immobilized SrtA displayed excellent stability and recyclability in this application.

### 2.5. Immobilized SrtA-Mediated Peptide Cyclization and Its Scale-Up Capability

**Figure 6:**
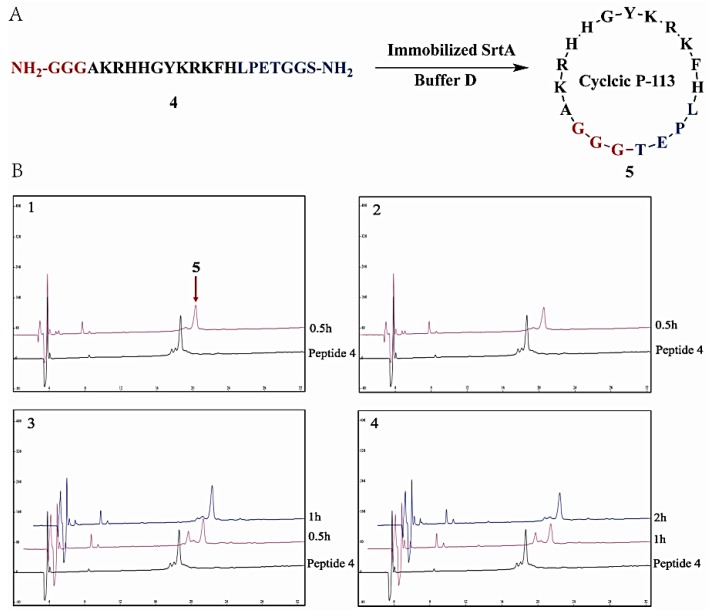
(**A**) Immobilized SrtA-mediated peptide cyclization of P-113; (**B**) HPLC diagraph of immobilized SrtA-mediated peptide cyclization of P-113 in different volumes; 1: 2 mL; 2: 10 mL; 3: 20 mL; 4: 40 mL. Experiments were performed in pH 7.5 at 37 °C as described in Section 3.6.2.

Sortase A-mediated ligation has been approved to be an efficient biocatalyst for the synthesis of cyclic peptide [13,52,53]. In these cases, the purification of the products from the reaction mixture is challenging. Additionally, recovering and recycling the biocatalyst is also very difficult. Therefore, to demonstrate the ability of immobilized SrtA in this application, an SrtA-mediated peptide head-to-tail cyclization reaction was performed by using antibacterial peptide P-113 as substrate [54]. The scale-up capability of using immobilized SrtA was also investigated in a batch reaction. As shown in Figure 6 and Table 5, when forty SrtA-immobilized particles were used in a 2 mL reaction volume with a peptide concentration of 0.2 mM, nearly a 100% conversion yield was observed in 30 min (Table 5, entry 1). When the reaction volume was scaled-up to 10 mL, approximately total conversion (99%) was achieved as determined by HPLC analysis (Table 5, entry 2). Moreover, when the reaction volume was enlarged to 20 mL and the amount of SrtA-immobilized chitosan beads was reduced by half, the reaction proceeded smoothly as well and the reaction completed in 1 h to give a conversion yield of 97% (Table 5, entry 3). Finally, when increasing the reaction volume to 40 mL, which contained approximately of 20 mg peptide **4**, the reaction was completed in 2 h and generated the cyclic peptide **5** in a yield as high as 95% (Table 5, entry 4). The conversion yield of the same cyclization reaction catalyzed by free SrtA was 93%. These results proved that the chitosan-immobilized SrtA retained excellent enzymatic activity and that the cyclization efficiency was comparable to that of free SrtA. In addition, the immobilized SrtA-mediated peptide head-to-tail cyclization could scale up to 40 mL without an obvious reduction in conversion yield, which may have great potential for industrial application.

## 3. Experimental Section

### 3.1. Materials and Methods

#### 3.1.1. Materials

Pre-constructed BL21 (DE3) harboring pET28a-△59SrtA (P94S/D160N/D165A/K196T, 4M) was used in this work [26]. Chitosan (90–95% deacetylated) was purchased from sangon Biotech Co., Ltd. (Shanghai, China). Glutaraldehyde was obtained from Sinopharm Chemical Reagent Co., Ltd. (Shanghai, China). All other regents were of analytical or HPLC grade.

#### 3.1.2. Expression and Purification of SrtA

An overnight seed culture of the strain mentioned above was inoculated into fresh TB (Terrific Broth) medium, incubated at 250 rpm and 37 °C until the OD_600_ reached 0.6. Protein expression was induced with 1 mM isopropyl-β-d-thiogalactoside (IPTG) at 30 °C for 4 h. Cells were harvested by centrifugation and resuspended in Buffer A (10 mM Tris-HCl, 0.5 M NaCl, pH 8.0). Then, cell disruption was performed on a vibrogen-cell mill (JXFSTPRP, Jingxin Industrial Development Co., Ltd., Shanghai, China) to obtain crude enzyme extract and the cell debris was removed by centrifugation at 9000 rpm at 4 °C for 10 min. With further purification through Ni-chelating affinity chromatography and salts removed by filtration, pure recombinant His-tagged SrtA was finally obtained. It was freeze-dried and stored at −20 °C for further use.

#### 3.1.3. Preparation of Chitosan Particles

Chitosan particles were prepared by the neutralization method [55]. A certain amount of chitosan powder was mixed with distilled water containing 0.3 M lactic acid, stirred, and heated until the chitosan powder was completely dissolved. Then, the solution was subjected to ultra-sonication to remove air bubbles. Chitosan particles were obtained by adding the resulting solution dropwise to a solution of 2 M NaOH with constant stirring and hardening for 1 h. The spherical particles formed with a diameter of 2–3 mm were thoroughly washed with distilled water until a neutral pH was reached and then stored at 4 °C for further use.

#### 3.1.4. Immobilization of SrtA

**Figure 7:**
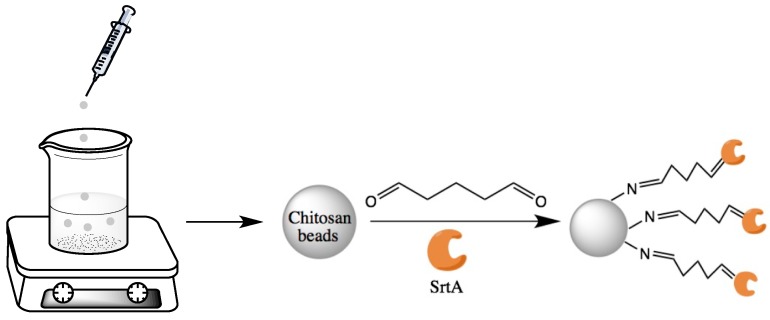
Scheme of preparation of chitosan particles and SrtA immobilization.

Before SrtA immobilization, ten chitosan particles were activated with 0.5 mL of diluted glutaraldehyde solution. After activation, excess glutaraldehyde was removed by washing extensively with buffer B (50 mM Tris, pH 8.0). Then, the activated particles were incubated with 0.5 mL of varied concentrations of SrtA; the supernatant after immobilization was removed for unbounded protein determination, and the particles were thoroughly washed with buffer B. After immobilization, the unreacted aldehyde group was blocked by excessive alanine at 20 °C for 2 h and the SrtA-immobilized chitosan particles were washed five times with buffer B to remove excess alanine. Figure 7 below summarized the preparation process of chitosan immobilized SrtA.

#### 3.1.5. Chitosan Particles Characterization

Fourier transform infrared spectroscopy (FTIR) of three kinds of chitosan particles were acquired by averaging 28 scans in the range of 4000–400 cm^−1^ with 4 cm^−1^ resolution (NEXUS, Thermo Nicolet Corporation, Waltham, MA, USA). Samples were lyophilized in advance; measurement of the sample before glutaraldehyde activation was performed using an attenuated total reflectance (ATR) sampling accessory because of the difficulty to pulverize, while the other two samples were measured by pressing to form transparent KBr pellets.

The detailed surface morphology of normal chitosan particles, glutaraldehyde-treated particles, and SrtA-immobilized particles was observed by scanning electron microscopy (SEM). Samples were freeze-dried previously and SEM imaging of these samples was carried out using Quanta-200 (FEI Corporation, Eindhoven, The Netherlands).

### 3.2. Experimental Design and Data Analysis

#### 3.2.1. Plackett–Burman Experimental Design

Plackett–Burman design (PBD) was applied to identify the operating variables that have a significant effect on SrtA immobilization. Seven parameters that may affect SrtA immobilization, including chitosan concentration (3–4%, *w*/*v*), glutaraldehyde concentration (0.5–2.5%, *v*/*v*), crosslinking temperature (20–30 °C), crosslinking time (0.5–3 h), SrtA concentration (0.457–1.371 mg·mL^−1^), immobilization temperature (4–20 °C), and immobilization time (2–12 h) were chosen at a high (+) and a low (−) level for screening. Responses were measured as specific activity of SrtA. The regression coefficient, effect values, *F* value, and *p*-values were determined using Design Export software@ 8.0.6.

#### 3.2.2. Response Surface Methodology (RSM)

The significant factors screened by PB design were studied in detail using a three-factor and three-level Box–Behnken design of the response surface methodology. Specific activity was recorded as the response and analyzed via Design Export software@ 8.0.6.

### 3.3. Activity Assays of SrtA

The activities of free and immobilized SrtA were measured using Dabcyl-QALPETGEE-Edans as substrate. When this peptide is cleaved between Thr and Gly by SrtA, the increment of fluorescence can be detected at an excitation wavelength of 350 nm and an emission wavelength of 495 nm. Fluorescence was monitored with a microplate reader (Synergy™ H4, Bio-Tek, Winooski, VT, USA). Generally, 40 μL of substrate (1 mg·mL^−1^), reaction Buffer C (50 mM Tris, 150 mM NaCl, 5 mM CaCl_2_, pH 7.5), and free enzyme or immobilized enzyme were mixed together to obtain a final volume of 0.5 mL, then the reaction system was incubated at 37 °C for 1 h to detect the fluorescence value. One unit of SrtA activity was defined as the amount of enzyme that catalyzes the formation of one fluorescence increment per minute. Specific parameters were calculated according to the following equations:

(1) $$\mathrm{Total}\;\mathrm{activity}\;\left(U\right)=\;\frac{I_{60}-I_{0}}{60}$$

(2) $$\mathrm{Specific}\;\mathrm{activity}\;\left(U\cdot {\mathrm{mg}}^{-1}\right)=\;\frac{I_{60}-I_{0}}{60\times m}$$

where *I*_60_ is the fluorescence intensity after the reaction, *I*_0_ is the fluorescence intensity of a blank control, and *m* (mg) is the protein content of SrtA.

(3) $$\mathrm{Activity}\;\mathrm{retention}\;\left(\mathrm{AR},\;\%\right)\;=\;\frac{\mathrm{specific}\;\mathrm{activity}\;\mathrm{of}\;\mathrm{immobilized}\;\mathrm{SrtA}}{\mathrm{specific}\;\mathrm{activity}\;\mathrm{of}\;\mathrm{free}\;\mathrm{SrtA}}\times 100$$

### 3.4. Determination of Protein Concentration

The protein content before or after immobilization was assayed by the Bradford method. Bovine serum albumin (BSA, Beyotime Biotechnology Co., Ltd., Shanghai, China) was used as the standard.

(4) $$\mathrm{Loading}\;\mathrm{efficiency}\;\left(\text{\%}\right)\;=\frac{C_{i}-C_{r}}{C_{i}}\times 100$$

where *C_i_* is the initial protein concentration of SrtA and *C_r_* is the protein concentration of SrtA after immobilization.

### 3.5. Stability of Free and Immobilized SrtA

#### 3.5.1. pH Stability

The pH stability of free and immobilized SrtA was investigated by incubation at 25 °C in a pH ranging from 5 to 10 for 30 min. Enzyme activity was determined as described above. The maximum activity was defined as 100%.

#### 3.5.2. Thermal Stability

The thermal stability of free and immobilized SrtA was investigated by incubation in buffer B at temperatures of 30, 40, 45, and 50 °C for 30 min. Enzyme activity was determined as described above. The maximum activity was defined as 100%.

### 3.6. Application of Immobilized SrtA in Peptide-to-Peptide Ligation and Peptide Cyclization

#### 3.6.1. Immobilized SrtA-Mediated Peptide-to-Peptide Ligation

Peptide **1** (Bz-LPETGGGS, 0.5 mM), peptide **2** (GGGGLA, 2.5 mM), and ten SrtA-immobilized chitosan particles were mixed in buffer D (300 mM Tris, 150 mM NaCl, 10 mM CaCl_2_, 2 mM mercaptoethanol, pH 7.5) at 37 °C for 1 h in 200 μL volume. Quantification of ligation product was determined by RP-HPLC using a C18 column with a linear gradient of 10–90% acetonitrile (0.1% TFA) over 20 min at a flow rate of 1 mL·min^−1^. The conversion yield was calculated based on the peptide peak area. Ligation product **3** was identified by MALDI-TOF-MS.

For the recycling and reuse of the immobilized SrtA: chitosan-immobilized SrtA was separated by filtration followed by washing with 2 mL of buffer B for five times. Then, it was used for the new batch reactions.

#### 3.6.2. Immobilized SrtA-Mediated Peptide Cyclization

Peptide **4** (NH_2_-GGGAKRHHGYKRKFHLPETGGS-NH_2_, 0.2 mM) and a certain amount of SrtA-immobilized chitosan particles were mixed in buffer D (0.3 M Tris, 0.15 M NaCl, 10 mM CaCl_2_, 2 mM mercaptoethanol, pH 7.5) at 37 °C in a defined volume. The cyclization process was monitored by RP-HPLC using a C18 column with a linear gradient of 10–28% acetonitrile (0.1% TFA) over 30 min at a flow rate of 1 mL·min^−1^. The resulting peak was confirmed by MALDI-TOF-MS.

## 4. Conclusions

In this study, we have prepared chitosan macro-particles by the neutralization method and they were applied to Sortase A immobilization using glutaraldehyde as a crosslinking agent. The immobilization process was optimized by response surface methodology and an average specific activity of 3142 U (mg protein)^−1^ was obtained under optimized immobilization conditions (SrtA concentration 0.5 mg·mL^−1^, glutaraldehyde concentration 0.5%, and immobilization time 8 h). The immobilized SrtA was successfully applied in a peptide-to-peptide ligation and reused for five cycles without obvious activity loss. Moreover, we demonstrated that immobilized SrtA was able to catalyze a head-to-tail peptide cyclization with excellent conversion efficiency and that the reaction could scale-up to 40 mL without an obvious reduction in conversion yield. The low-cost, biocompatible, and simple preparation protocol will make chitosan-immobilized SrtA have great potential for industrial applications.

## Figures and Tables

**Table 1:** Regression analysis for the selected Plackett–Burman (PB) model.

	Effect Value	*F* Value	Prob > *F*	Value
A	−16.29	0.18	0.6949	-
B	−345.78	80.14	0.0009	-
C	−75.89	3.86	0.1209	-
D	92.75	5.77	0.0743	-
E	−211.76	30.06	0.0054	-
F	110.39	8.17	0.0460	-
G	164.69	18.18	0.0130	-
Model	-	20.91	0.0053	-
R^2^	-	-	-	0.97
Adj R^2^	-	-	-	0.93
Pred R^2^	-	-	-	0.76

A: concentration of chitosan; B: concentration of glutaraldehyde; C: crosslinking temperature; D: crosslinking time; E: concentration of SrtA; F: immobilization temperature; G: immobilization time.

**Table 2:** Experimental levels of variables in the Box–Behnken experimental design.

	A (%)	B (mg·mL^−1^)	C (h)
Low level (−1)	0.2	0.2	4
Central level (0)	0.5	0.5	8
High level (+1)	0.8	0.8	12

A: Glutaraldehyde concentration; B: SrtA concentration; C: Immobilization time.

**Table 3:** Box–Behnken design of experiments for 17 trial runs.

Entry	A (%)	B (mg·mL^−1^)	C (h)	Total Enzyme Activity	Binding Protein (mg)	Loading Efficiency (%)	Specific Activity (U·mg^−1^)	Activity Retention (AR) (%)
1	−1	0	+1	97.55	0.098	39.2	998.65	3.21
2	0	0	0	588.28	0.199	79.6	2952.47	9.50
3	−1	0	−1	79.05	0.067	26.8	1172.03	3.77
4	−1	+1	0	167.93	0.151	37.8	1115.51	3.59
5	0	−1	+1	165.85	0.100	100	1658.50	5.33
6	+1	0	+1	346.48	0.250	100	1385.93	4.46
7	0	0	0	550.10	0.185	74	2971.93	9.56
8	0	+1	+1	849.78	0.309	77.3	2751.93	8.85
9	0	−1	−1	276.50	0.085	85	3242.40	10.43
10	0	0	0	637.48	0.185	74	3438.04	11.06
11	−1	−1	0	53.75	0.037	37	1451.57	4.67
12	0	+1	−1	665.40	0.255	63.8	2611.89	8.40
13	+1	0	−1	450.12	0.221	88.4	2035.59	6.55
14	+1	+1	0	599.80	0.356	89	1686.33	5.42
15	+1	−1	0	170.65	0.099	99	1721.87	5.54
16	0	0	0	592.40	0.182	72.8	3251.30	10.46
17	0	0	0	572.47	0.185	74	3098.15	9.96

**Table 4:** ANOVA of response surface methodology (RSM) variables for specific activity of immobilized SrtA.

	*F* Value	Prob > *F*	Value	
Model	27.75	0.0001	-	Significant
A	11.84	0.0108	-	-
B	0.023	0.8848	-	-
C	13.90	0.0074	-	-
AB	0.49	0.5071	-	-
AC	1.23	0.3045	-	-
BC	16.08	0.0051	-	-
A^2^	180.52	<0.0001	-	-
B^2^	5.22	0.0563	-	-
C^2^	10.23	0.0151	-	-
R^2^	-	-	0.9727	-
Adj R^2^	-	-	0.9377	-
Pred R^2^	-	-	0.7673	-
Lack of fit	1.25	0.4040		Not significant

**Table 5:** Chitosan-immobilized SrtA-mediated P-113 cyclization.

Entry	Volume (0.48 mg/mL^−1^)	Reaction Time (h)	Number of Beads	Yield (%)
1	2 mL	0.5	40	100
2	10 mL	0.5	200	98.5
3	20 mL	1	200	97.3
4	40 mL	2	400	95.4

## Supplementary Materials

Supplementary materials are available online. Figure S1 (MALDI-TOF mass spectrum of peptide **1**, **2** and **3**) and Figure S2 (MALDI-TOF MS of peptide **4** and **5**).
