# Supplementary material for: Immobilization of Staphylococcus aureus Sortase A on Chitosan Particles and Its Applications in Peptide-to-Peptide Ligation and Peptide Cyclization
Source: Molecules. 2018 Jan 19;23(1):192. doi: 10.3390/molecules23010192 (PMC6017383; doi:10.3390/molecules23010192)
Supplement: Supplementary file 1 [file molecules-23-00192-s001.pdf]

Supplementary Materials for

Immobilization of *Staphylococcus aureus* Sortase A on Chitosan Particles and Its Application in Peptides Cyclization

Min Yang, Haofei Hong, Shaozhong Liu, Xinrui Zhao, Zhimeng Wu*

Key Laboratory of Carbohydrate Chemistry & Biotechnology, Ministry of Education, School of Biotechnology, Jiangnan University, Wuxi, 214122, China; 18262281871@163.com (M.Y.); hhf_1992@163.com (H.H.); liushaozhong90@163.com (S.L.); zhaoxinruibs0511@163.com (X.Z.)

* Correspondence: zwu@jiangnan.edu.cn; Tel.: +86-510-85197582

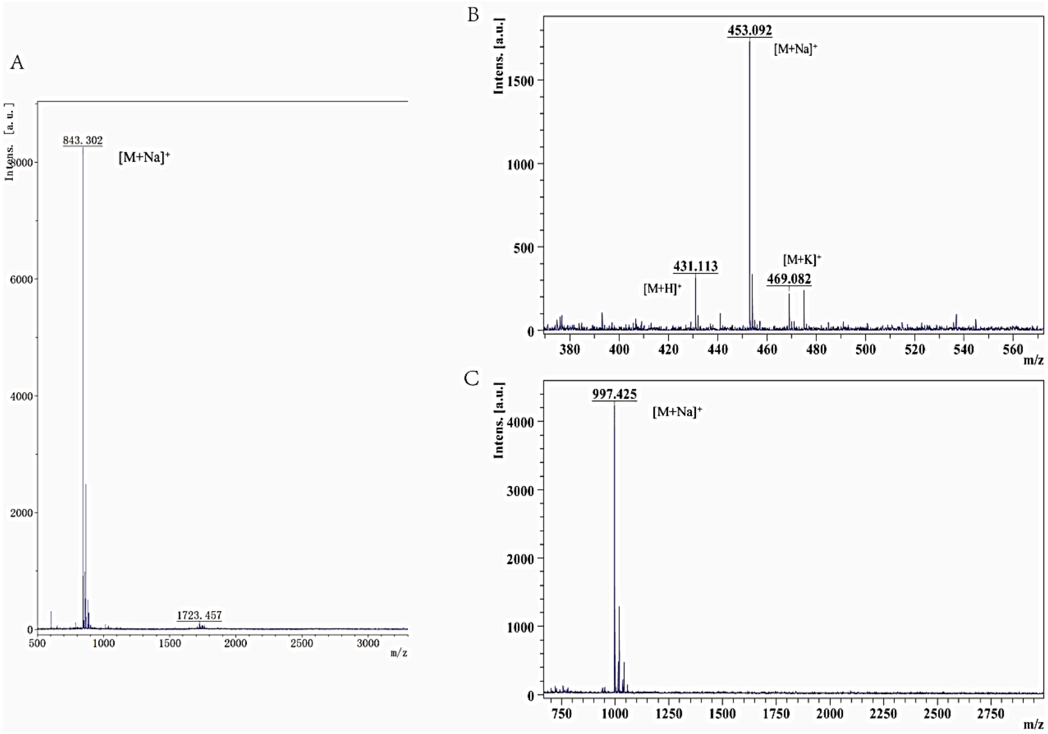

Fig.S1. (A) MALDI-TOF mass spectrum of peptide **1**, calcd for 820.86, observed 843.3. $[M+Na]^+$ (B) MALDI-TOF mass spectrum of peptide **2**, calcd for 430.46, observed 431.111 $[M+Na]^+$, 453.093 $[M+Na]^+$, 469.066 $[M+K]^+$. (C) MALDI-TOF mass spectrum of peptide **3**, calcd for 975.07, observed 997.425 $[M+Na]^+$.

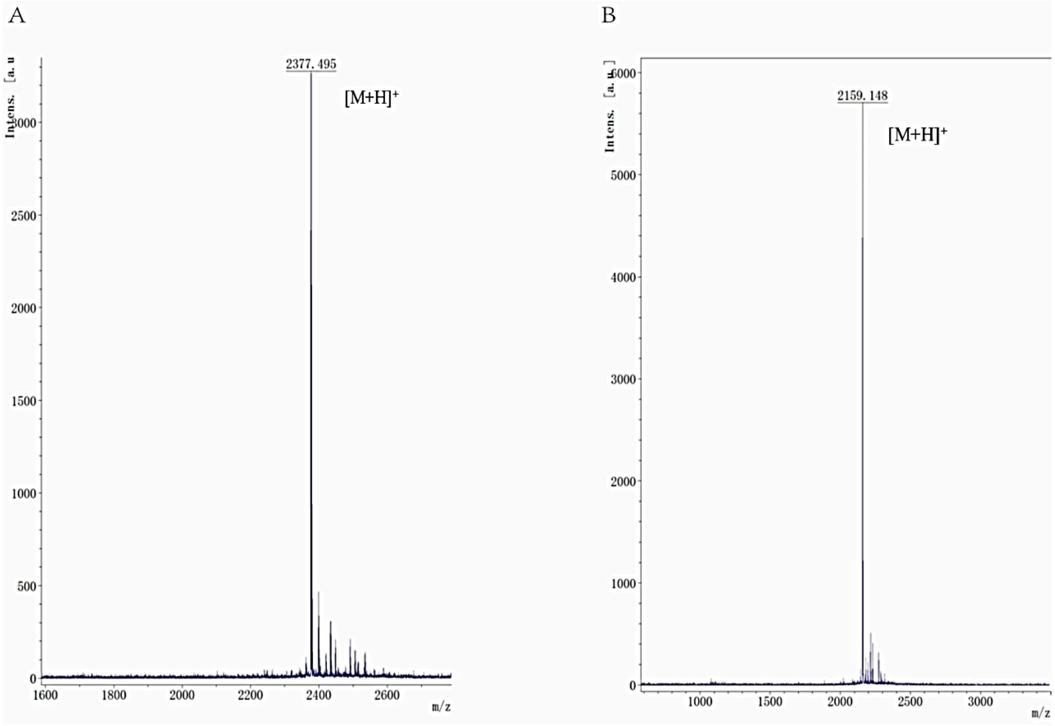

Fig.S2. MALDI-TOF MS of (A) Peptide **4**, calcd for 2376.6, observed 2377.5 [M +H]$^{+}$. (B) Peptide **5**, calcd for 2158.4, observed 2359.1 [M + H]$^{+}$.
